# Sufentanil, dexmedetomidine combined with surface anesthesia for awake fiberoptic nasotracheal intubation in the patient with severe mouth opening difficulty undergoing wedge resection of the right upper lung: A case report and literature review

**DOI:** 10.1097/MD.0000000000033584

**Published:** 2023-04-21

**Authors:** Yanwei Zhang, Xiang Lv, Pingping Sun, Dekun Yin

**Affiliations:** a Department of Anesthesiology, Shanghai Ninth People’s Hospital, Shanghai Jiao Tong University School of Medicine, Shanghai, China; b Department of Anesthesiology and Intensive Care Units, Funing People’s Hospital of Jiangsu, Yancheng, Jiangsu province, China.

**Keywords:** AFNI, dexmedetomidine, medication scheme, mouth opening difficulty, sufentanil, topical anesthesia

## Abstract

Severe mouth opening difficulty may increase the risk of airway management during anesthesia induction, and awake fiberoptic nasotracheal intubation (AFNI) is the first option for patients with orofacial anatomical changes.

**Case summary:**

A 54-year-old man was scheduled to undergo wedge resection of the right upper lung in August 2021. The patient had a history of enlarged right maxillary lesion resection and partial right maxillary resection surgery in April 2020, which led to orofacial anatomical changes and severe mouth opening difficulty. To avoid difficult airway-related emergency scenarios, the AFNI was successfully performed through intravenous injection of sufentanil and dexmedetomidine combined with lidocaine topical anesthesia under a conscious state without any uncomfortable feeling or complications.

**Conclusions:**

Intravenous injection of sufentanil and dexmedetomidine combined with lidocaine topical anesthesia can be used as an alternative medication scheme to relieve uncomfortable suffering for AFNI in patients with severe mouth opening difficulty.

## 1. Introduction

For patients with malignant oral neoplasm who have received surgery and chemoradiotherapy, the upper airway structural changes and concomitant severe mouth opening difficulty have become the most common complication, caused by a series of progressive physiological and pathological changes in the surgery area, such as inflammatory reaction, desmoplasia, focal adhesions, and tissue remodeling, deposition of calcium salts within the tissue, and bony hyperostosis.^[1]^ Hence, airway management in such patients is of prime importance, and orotracheal double-lumen endotracheal intubation is not a good choice when they accept the other procedures. General anesthesia with a double-lumen tube (DLT) is the conventional norm for thoracoscopic procedures to achieve complete operated-lung collapse and contralateral-lung ventilation, which can effectively block blood, infectious substances, and even tumor cells entering the contralateral-lung.^[2]^ To achieve the goals of the nasal cannula and pulmonary sequestration, awake fiberoptic nasotracheal intubation (AFNI) combined with a bronchial blocker is considered a safe and effective alternative approach for the patient with oral surgery-induced severe mouth opening difficulty.^[3]^ Regrettably, awake fiberoptic nasotracheal intubation causes great physical and mental suffering to the patients, which also increases the rates of respiratory complications such as epistaxis, persistent nasal discharge, coughs, and asphyxia. Overuse of opioids and/or sedations also poses huge risks of apnea and oxygen supply difficulty to individual patients. However, the guiding medication regimes for AFNI are seldom reported in the literature. This article reports a combinatorial treatment protocol for AFNI, which mainly includes sedation, analgesia, and surface anesthesia. The possibility of nasal destruction and hemorrhage simultaneously be prevented by vasoconstrictors and lubricants. The effective medication regimes of this case have important guiding value for clinical management.

## 2. Case description

**Figure 1.**
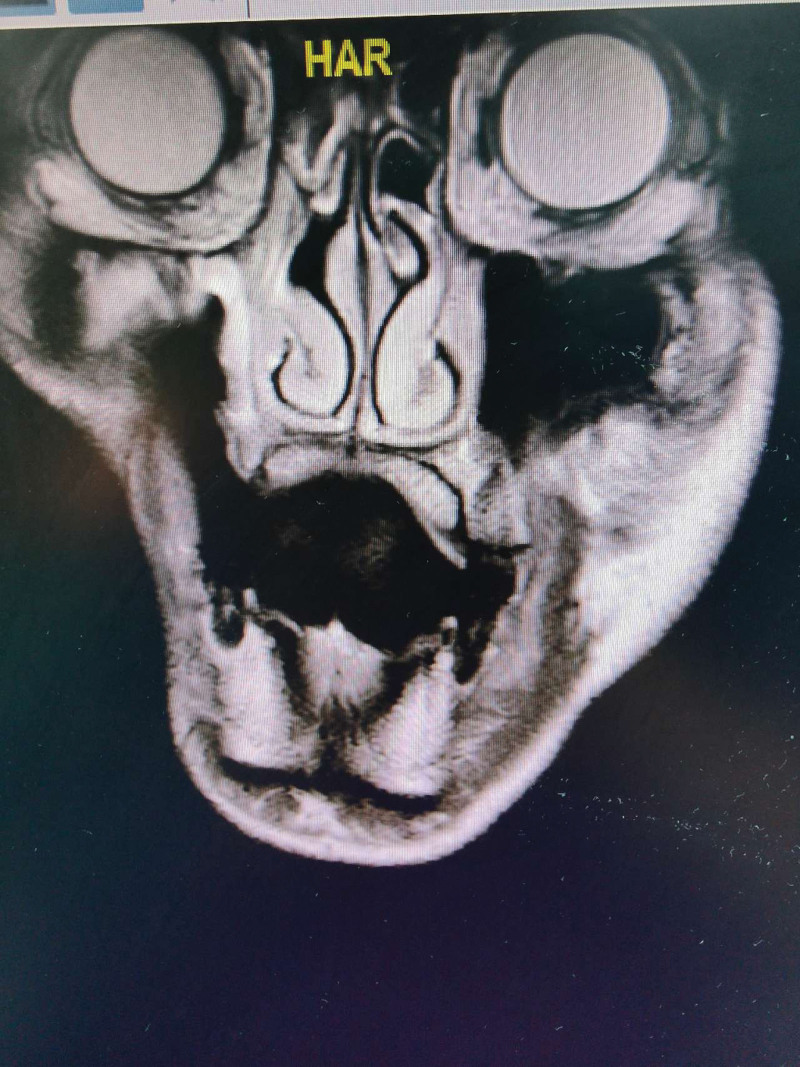
CT showing the patient’s right maxillary resection and anatomical changes of the upper airway. CT = computed tomography.

**Figure 2.**
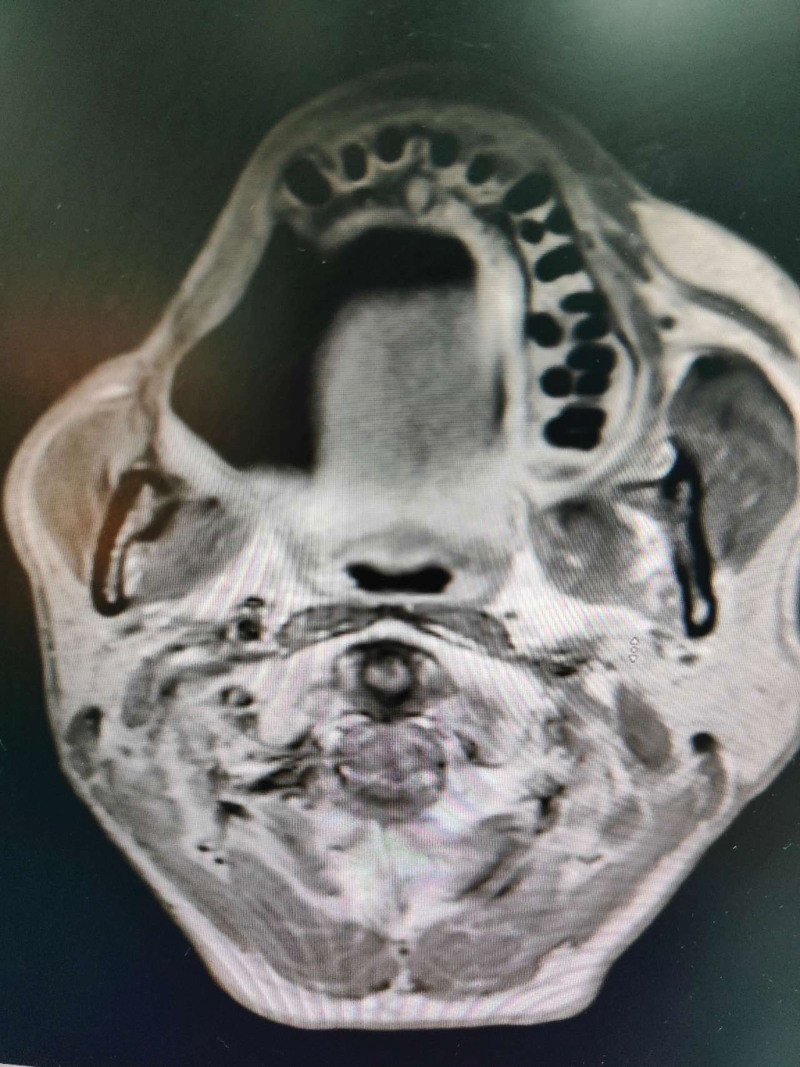
CT scan revealed a partial absence of the right mandible.

**Figure 3.**
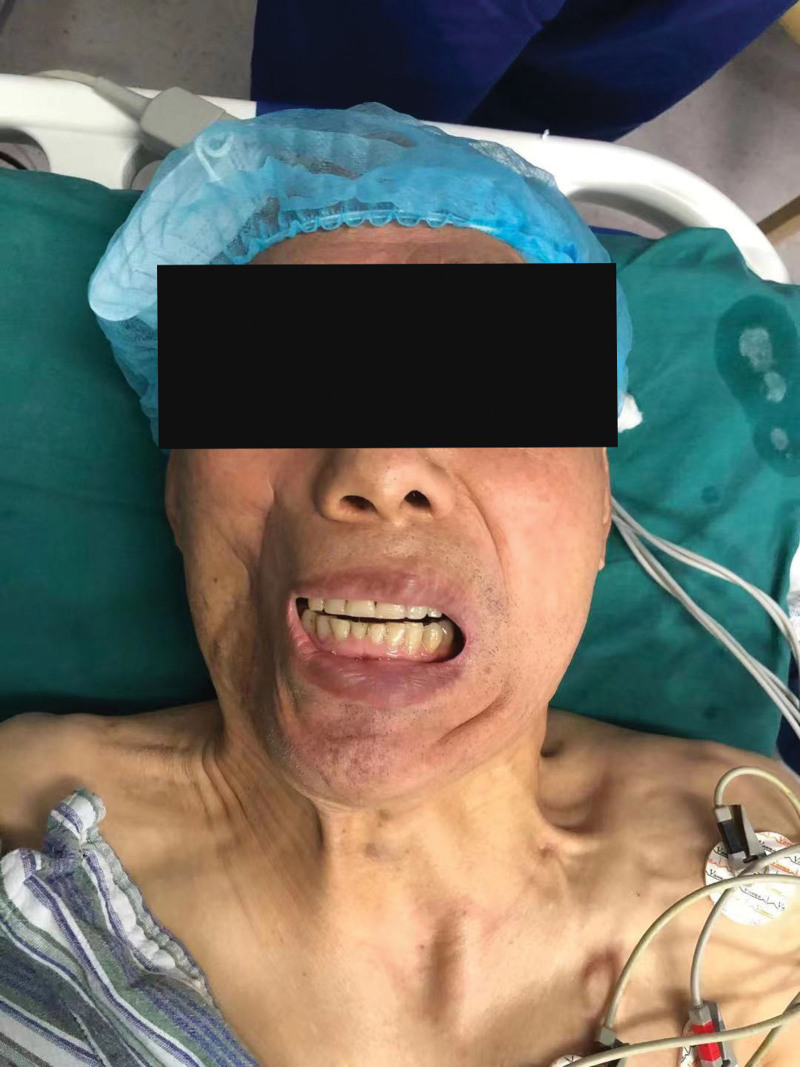
The patient was completely unable to open his mouth after oral surgery treatment.

A 54-year-old man (height, 174 cm; weight, 70 kg) was scheduled to undergo wedge resection of the right upper lung and closed thoracic drainage due to multiple pulmonary nodules in August 2021, which probably represented the malignant disease in the upper lobe of the right lung through preoperative evaluation. In April 2020, the patient underwent oral surgery because of right maxillary angiosarcoma, including enlarged right maxillary lesion resection, partial right maxillary resection, and adjacent flap metastasis repair (Figs. 1 and 2). After surgery, the patient received chemoradiotherapy and has an excellent outcome without any signs of recurrent disease. He, therefore, presented with severe mouth opening difficulty, maxillary retrusion, and facial asymmetry, indicating a difficult airway (Mallampati classification IV, mouth opening < 3 cm) (Fig. 3). Chest computed tomography/X-ray demonstrated multiple nodules in the upper lobe of right lung, while no abnormalities were found in the pulmonary function examination. An electrocardiogram showed that there was a normal sinus rhythm with premature ventricular contraction, and left deviation axis. The results of other laboratory tests were within the acceptable range including blood, urine, and so on. In addition, the patient had well-controlled hypertension by using the drugs metoprolol and nifedipine, and no alcohol/tobacco use.

**Figure 4.**
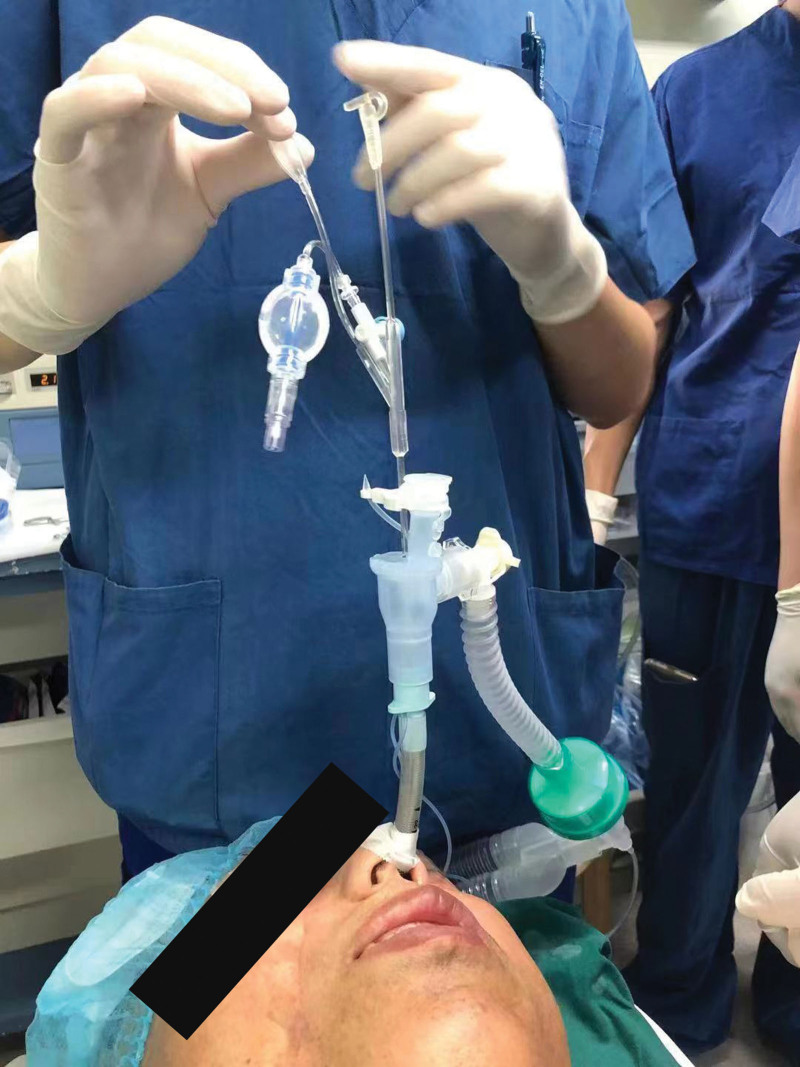
Bronchoscopy successful nasotracheal intubation with branchial blocker in patient with restricted patient mouth opening.

Before surgery, anesthesia scenarios (potential impending dangers and response measures, etc) were explained to the patient and his family, then they agreed and signed informed consent forms for anesthesia. After entering the operating room and establishing peripheral venous access, vital signs were monitored including 5-lead electrocardiography, pulse oximetry, and invasive blood pressure. The oxygen was administered by face mask. Considering the patient with severe mouth opening difficulty, ANFI was recommended as the optimal choice for anesthetists. To avoid discomfort, distress, pain, and injury for the patient, we developed the following strategy, and remarkable results had been achieved. First, the patient was injected with dolasetron (12.5mg) and penehyclidine hydrochloride (0.5mg) 30 minutes prior to surgery. A few minutes later, sufentanil (10 ug) was given via intravenous injection, and a continuous infusion of dexmedetomidine (0.1 ug/[kg·minutes]) lasted for 10 minutes. Second, ephedrine and furacilin nasal drops were dripped into the left nasal cavity to prevent epistaxis. There 3 mL of lidocaine 2% was administered intratracheally through cricothyroid membrane puncture, and the patient was asked to cough to ensure full diffusion. Lidocaine hydrochloride spray was also used for regional anesthesia in the vocal cords, supraglottal region, laryngeal region, posterior pharynx, and left nasal cavity. After repeating 3 times for adequate topical anesthesia, nasopharyngeal secretions and medicine liquids were removed by gentle aspiration. The paraffin oil was then dripped into the left nasal cavity for a few drops, and it was also coated on the surfaces of the fiberoptic bronchoscopy (FOB) and tracheal tube. Third, to facilitate accurate positioning of the FOB, transpharyngeal axis, and translaryngeal axis should be close to overlapping through head-up and chin-lift by an assistant. After the endotracheal tube (ETT) being sleeved on the fiberoptic bronchoscope, they were inserted in the trachea along the nasal cavity, throat and vocal cord. Finally, the fiberoptic bronchoscope was completely removed, and the correct positioning of the tracheal tube was confirmed by monitoring of end-tidal carbon dioxide when the ETT (ID = 7.0) was connected to the breathing circuit after the cuff being inflated. The patient was determined to be pain-free, and propofol (100 mg) and rocuronium (30 mg) were then given via intravenous injection. After 3 minutes, a bronchial blocker was placed under the guidance of the FOB to enable the occlusion of the right tracheal bronchus (Fig. 4). In parallel, the left radial artery puncture catheterization was conducted to monitor the continuous arterial pressure and the minimally invasive cardiac output. The operation lasted approximately 1 hour under stable circulation with stable vital signs. Following the operation, the patient was admitted and monitored in the intensive care unit overnight, and the ETT was removed to resume spontaneous respiration on the second postoperative day. When being followed up after several days of recovery, informed consent was obtained from the patient for publication of this case report details.

## 3. Discussion

Angiosarcoma is a rare and aggressive malignant tumor originating from vascular and lymphatic endothelial cells, accounting for only 1 to 2% of all soft-tissue sarcomas,^[4]^ but only 4% of angiosarcoma cases occur in the pharynx, oral cavity and paranasal sinuses.^[5]^ Regardless of the pathology-prognostic grade, oral angiosarcoma exhibits a relatively better prognosis compared with other angiosarcomas, which is known to closely correlate with the invasive and metastatic properties of tumor cells.^[6]^ For the patient with a history of intraoral, pharyngeal and neck tumors, anesthetic airway management would be very difficult when accepting the other surgery because of the pathophysiologic/anatomical structural changes induced by surgical resection, radiotherapy and chemotherapy. In this case, the patient was recognized as difficult airway through preoperative evaluation according to maxillary retrusion, facial asymmetry, and Mallampati classification IV, mouth opening of <1.5 cm. The regimen of AFNI should be prioritized in this patient with mouth opening limitation.

With the rapid development of thoracic surgery technology and the popularity of the enhanced recovery after surgery concept, the 2-minute disconnection technique with the DLT or bronchial blocker not only offers an effective method to accelerate the collapse of the non-ventilated lung during 1-lung ventilation for thoracoscopic surgery, but also would be effective to enhancing prognosis, and reducing complication for the patients who have severe destructive lung parenchyma, hemothorax, pleural effusion, lung neoplasms.^[2]^ However, different from bronchial blocker, the DLT hold advantages in avoiding intraoperative and perioperative complications, such as contralateral-lung tumor implantation.^[2]^ Furthermore, ventilation switching between the 1-lung and 2-lung is simple, fast, reliable and secure after double-lumen endotracheal intubation.^[2]^ The DLT is also conducive to removing the blood or secretions in operated-lung before 2-lung ventilation in order to reduce airflow resistance without affecting gas exchange in contralateral-lung ventilation.^[2]^ The above advantages cannot be achieved by bronchial blocker. Therefore, based on the reliability of lung isolation and the convenience of tube localization, a double-lumen endobronchial tube is considered to be the optimal choice for achieving contralateral-lung ventilation and operated-lung collapse during open-chest surgery in order to maintain stable vital signs and providing a good surgical view and sufficient space.^[2,7]^ Regrettably, the large size and curvature of double-lumen tubes lead to various intubation complications such as intubation difficulty, damage to the glottis, throat edema and postoperative hoarseness. Absolute and relative contraindications for orotracheal intubation by double-lumen tube include mouth opening limitation (Mallampati 3 or 4), herniated upper incisor, retraction of the mandible, restricted cervical spine movement, history of head and neck trauma, radiotherapy or tumor resection and so on.^[7]^ The 1-lumen tube combined with a bronchial blocker via the nasal route of intubation therefore becomes a better choice for this patient with severe mouth opening difficulty induced by the upper respiratory tract anatomical structure changes, which make it difficult to implement orotracheal intubation. Although locating difficultly and intraoperative repositioning frequently, previous reports indicated that lung collapse was more rapid and thorough by using bronchial blockers, and it also was beneficial for improving oxygenation and removing carbon dioxide through selective lobar blockade to increasing the gas exchange area.^[8,9]^ Moreover, compared endotracheal tube (EZ) blockers and left double-lumen tube in 100 patients, ETT blockers were considered even more concise and easy to operate, with a lower incidence of complications such as postoperative sore throat, tract injury, tracheal or bronchial hematoma.^[10,11]^ In brief, DLTs are easier and faster to insert than bronchial blockers, which are associated with more side effects. However, there was no significant difference in the quality of lung isolation offered by the 2 devices, and both approaches showed advantages in certain clinical conditions.^[12]^

Due to an anticipated difficult airway, awake fibrotic endotracheal intubation is performed under surface anesthesia combined with sedatives and analgesics in clinical practice, which effectively reduces patients discomfort. However, the rate of complications induced by nasal intubation remains high: tracheal mucosal laceration, hemorrhage, edema, pressure-induced ischemic injury, and stricture formation. In the literature, there are also few studies presenting details of the implementation and right medication regimen for conscious sedation with local anesthesia. In this case report, the medication regimen and implementation steps are described in detail and can avoid all of these problems above, which has a certain guiding significance for the clinical application of awake intubation. As we know, dolasetron can be effective for the relief of nausea and vomiting. As a preanesthetic medication, penehyclidine hydrochloride can not only effectively reduce respiratory secretions and vascular infiltration but also strengthen sedation, and bidirectionally regulate heart rate to keep heart rate constant.^[13]^ Obviously, the ideal target for sedation during awake fiberoptic intubation is that the patient can be provided the awake intubation without any comfortlessness and potential risks, such as airway obstruction, respiratory depression, cough reflex, airway hyperreactive response, secondary hemodynamic changes, and related complications.

As a selective α-2-adrenergic receptor agonist, dexmedetomidine has the effects of analgesic and sedation without suppressing the respiratory system, which may be beneficial for patients with difficult or unstable airways.^[14]^ Different from other sedatives, dexmedetomidine may have a relatively higher degree of safety because of its bronchial relaxing effect and reducing oral secretions.^[15]^ Moreover, compared to improving the dosimetric, dexmedetomidine combined with low-dose fentanyl (1μg/kg) have a significantly reduced risk of airway obstruction, while obtaining the same favorable intubation score. Therefore, the patient received a standardized treatment regimen of dexmedetomidine combined with low-dose sufentanil. Although a study in Chinese hospitals had concluded with findings that well-lubricated nasotracheal intubation does not require pretreatment with ephedrine to reduce prevent nasotracheal intubation-related epistaxis, the vasoconstrictor drugs (ephedrine and furacilin) had been administered for the patient to avoid edema formation induced by tracheal tube extrusion at the stenosis of oral and nasal. According to clinical experiences, single oronasal surface anesthesia does not completely block the stress response (especially choking reflex and glottis closure state), and airway surface anesthesia is also implemented through cricothyroid membrane puncture to eliminate undesirable reflections sufficiently. In this case, different anesthesia methods could be compounded or combined and supplemented by various drugs or means to inhibit the stress response, and the patient was well tolerated throughout the awake fibrotic endotracheal intubation without any obvious cardiovascular complications.

In the present case study, we report a standardized medication scheme to relieve uncomfortable suffering and to achieve intubation successfully without any injury for awake nasal fiberoptic bronchoscopic tracheal intubation in patients with severe mouth opening difficulty, including antiemetic drugs, secretion inhibitors, sedative and analgesic drugs, vasoconstrictor drugs, oronasal and tracheal surface anesthesia, lubricant.

## Acknowledgments

We appreciate the hospital staff for their support and assistance and thank for the understanding and cooperation of participants.

## Author contributions

**Project administration:** Yanwei Zhang, Dekun Yin.

**Software:** Pingping Sun.

**Supervision:** Dekun Yin.

**Writing – original draft:** Yanwei Zhang, Xiang Lv, Pingping Sun.

**Writing – review & editing:** Dekun Yin.
